# Nicotine consumption and folate insufficiency in pregnancy: a population-based cross-sectional study

**DOI:** 10.1080/14767058.2025.2577231

**Published:** 2025-10-27

**Authors:** Weiyi Huang, Melissa B. Harrell, Robin L. Page, Theresa Morris, Susan Ayres, Mahua Choudhury, Dayana Betancourt, Samiran Sinha

**Affiliations:** a Department of Biostatistics and Data Science, University of Texas Health Science Center at Houston, Houston, TX, USA; b Department of Epidemiology, Michael & Susan Dell Center for Healthy Living, School of Public Health, University of Texas Health Science Center at Houston, Austin, TX, USA; c School of Nursing, Texas A&M University, College Station, TX, USA; d Department of Sociology, Texas A&M University, College Station, TX, USA; e School of Law, Texas A&M University, College Station, TX, USA; f Department of Pharmaceutical Sciences, Texas A&M University, College Station, TX, USA; g Birth Defects Epidemiology and Surveillance Branch, Texas Department of State Health Services, Austin, TX, USA; h Department of Statistics, Texas A&M University, College Station, TX, USA

**Keywords:** Cotinine, health disparities, pregnancy outcomes, red blood cells folate, tobacco smoking

## Abstract

**Objective:**

Tobacco smoking and folate insufficiency are both risk factors associated with adverse pregnancy outcomes, but their association in pregnancy remains unclear. This study investigated the association between tobacco smoking and folate insufficiency in pregnant women in the U.S.

**Methods:**

Data from nine consecutive cycles of the National Health and Nutrition Examination Survey (2003–2020) were analyzed. Smoking status was derived from serum cotinine levels, and folate insufficiency was determined based on World Health Organization guidelines. The Rao-Scott test of independence was used to assess the prevalence of smoking and folate insufficiency across sociodemographic subgroups, and survey-weighted logistic regression models were used to evaluate the association between smoking and folate insufficiency.

**Results:**

Both smoking and red blood cell (RBC) folate insufficiency showed high prevalence among non-Hispanic Black subgroups with an education level of high school or less. Compared to pregnant nonsmokers, pregnant smokers faced increased odds of RBC folate insufficiency (OR: 1.87; 95% CI: 1.10, 3.19). Approximately 3.6% (95% CI: 1.4, 6.4%) of cases of RBC folate insufficiency among pregnant women in the U.S. were associated with active smoking.

**Conclusions:**

Tobacco smoking increases the risk of insufficient folate stores among pregnant women. However, healthcare providers should assess the folate status of all pregnant women and consider proactive screening, such as RBC folate testing, complemented by integrated strategies addressing tobacco use and nutritional risk. Proactive screening for smokers should be revisited once the prevalence of insufficient folate stores has been reduced at the population level.

## Introduction

Folate, the natural form of Vitamin B9, is a water-soluble vitamin that is essential for DNA synthesis, DNA and histone methylation, hematopoiesis, and embryonic development. Folate deficiency in pregnancy affects the methylation patterns during embryonic development and genomic stability, causing neural tube defects (NTDs) in offspring [1]. While the mechanism behind NTDs is complex and multifaced involving genetics, chromosomal abnormalities, and environmental factors, studies have confirmed that folate deficiency is associated with the risk of birth with NTDs [2], and periconceptional folate supplementation has been shown to reduce the occurrence of fetal NTDs [3]. Even though the Food and Drug Administration (FDA) mandated the addition of folic acid to all enriched cereal-grain foods in 1998 to prevent folate deficiency in women of childbearing age and the occurrence of neural tube defects in their offspring, folate deficiency remains an issue among disadvantaged populations, such as ethnic minority and low-income women [4]. Addressing the issue of folate deficiency in pregnancy is important for decreasing birth defect rates, reducing health disparities, and achieving health equity in the U.S. Folate deficiency in pregnant women is best measured using red blood cell (RBC) folate levels, as, compared with serum folate levels, RBC folate levels are more reliable and reflect long-term folate stores which could be compromised by cigarette smoking [5].

Tobacco smoking is the most common source of nicotine exposure, and cotinine, as a biomarker of tobacco smoke exposure, is a nicotine metabolite [6]. Nicotine has various biological effects, such as gene expression, enzyme activity, and oxidative stress [7]. Nicotine and its metabolite cotinine can pass through the blood-placenta interface and be transported to the fetus, which is detrimental to fetal development [8]. Tobacco use in pregnancy has a causal link to adverse neonatal outcomes, including low birth weight, birth defects, and sudden infant death syndrome (SIDS), as well as to adverse pregnancy outcomes, such as miscarriage and stillbirth [9]. Despite the known risks, smoking during pregnancy remains a concern in the U.S., particularly for young women, American Indians/Alaska Natives, and women with a high school diploma [10]. Addressing the issue of tobacco consumption in pregnancy could help protect high-risk pregnant women and their offspring from adverse outcomes and lessen this health disparity.

Although previous studies have reported associations between folate deficiency in pregnancy and adverse pregnancy outcomes as well as between smoking during pregnancy and adverse outcomes, the association between folate status and nicotine consumption, along with the potential biological mechanisms involved, remains unclear. Folic acid has been shown to protect against nicotine-induced pancreatic β-cell apoptosis and damage by modulating oxidative stress and pro-inflammatory cytokine levels [11]. It is unclear whether folate’s protective effects against nicotine extend to fetal neurodevelopment. We analyzed data from a cross-sectional study to provide evidence for the association between folate status and nicotine consumption in pregnancy. We identified nicotine consumption status using serum cotinine concentration and identified folate insufficiency using World Health Organization (WHO)–recommended optimal NTDs prevention concentrations. This is the first study to use a nationally representative sample of the U.S. population to examine the relationship between smoking behavior and folate insufficiency in pregnancy, as well as to assess the prevalence of folate insufficiency based on WHO-recommended guidelines.

## Methods

We utilized cross-sectional survey data from nine consecutive survey cycles (2003–2020) of the National Health and Nutrition Examination Survey (NHANES), which collects data to generate samples representative of the U.S. population *via* a stratified and multistage probability cluster sampling method. During the period from 2003 to 2020, a total of 1027 women were confirmed pregnant by a rapid chromatographic immunoassay (urine pregnancy test). We excluded women who had a liver condition or had weak/failing kidneys or those on certain medications (such as Carbamazepine, Methotrexate, Phenytoin, and Trimethoprim) as these medical conditions and medications can cause folate deficiency or interfere with nicotine metabolism. A total of 780 pregnant women with complete information between the ages of 20 and 44 were included in this study.

Participants completed a home interview followed by a standardized health examination in a Mobile Examination Center (MEC), where blood samples *via* venipuncture and urine specimens (midstream clean-catch urine sample) were collected at a single visit [12]. Generally, blood samples are sent to CDS laboratories or partner labs for analysis but hematocrit measurements are done on-site. From the blood samples, folate concentrations are measured by microbiologic assay and isotope-dilution high-performance liquid chromatography coupled to tandem mass spectrometry (LC-MS/MS). The categorization of folate concentrations is based on evidence-based folate concentration guidelines by the WHO. Folate insufficiency in pregnancy is defined as red blood cell (RBC) folate concentration below the threshold of 400 ng/mL, which indicates suboptimal NTD prevention [13]. Serum cotinine is measured by an isotope dilution-high performance liquid chromatography/atmospheric pressure chemical ionization tandem mass spectrometry (ID HPLC-APCI MS/MS). Women with serum cotinine levels higher than 10 ng/mL are categorized as active smokers, women with levels <1 ng/mL are categorized as nonsmokers, and women with levels in the 1–10 ng/mL range are categorized as having heavy exposure to secondhand tobacco smoke [14].

The characteristics of women having cotinine-derived smoking status and folate insufficiency prevalence were compared by age, race/ethnicity, marital status, educational level, and family income. The Rao-Scott test of independence was used to test statistical differences, and *p* < 0.05 was considered statistically significant. The survey-weighted logistic regression model (a generalized linear model with binomial family and logit link) was used to estimate the association between RBC folate insufficiency and smoking exposure specified as (i) a three-level categorical variable (active smoker, heavy secondhand smoke exposure, nonsmoker) and (ii) a continuous serum cotinine measure scaled per 12.5 ng/mL, for both unadjusted and covariate-adjusted analyses. Odds ratios (ORs) and 95% confidence intervals were reported. Population attributable fractions (PAFs) [15,16] were used to measure the proportion of folate insufficiency events attributable to smoking status after adjustment of confounding variables, which represented the proportion of folate insufficiency events associated with tobacco smoke exposure. PAF is used to measure the public health impact of the exposure. The *E*-value measure was used to perform the sensitivity analyses for unmeasured confounding in our study [17]. Survey weights based on stratum and primary sampling unit were used in all analyses. All analyses and figures were performed using the statistical software R, version 4.3.2.

## Results

We analyzed the prevalence of folate insufficiency among pregnant women in the U.S. Overall, 47.2% had RBC folate insufficiency. Significant differences were observed in folate insufficiency prevalence based on race/ethnicity, educational level, and family income (Table 1). RBC folate insufficiency was most prevalent among non-Hispanic Black pregnant women and those with a high school education or less (both *p* < 0.05, Table 1). Additionally, we examined the smoking status of pregnant women in the U.S. Overall, 12.7% of pregnant women were active smokers, and 4.0% were heavily exposed to secondhand smoke. Significant variations in smoking status were found based on race/ethnicity, marital status, and educational level (all *p* < 0.05, Table 2). The highest percentages of active smokers and those heavily exposed to secondhand smoke were among non-Hispanic Black pregnant women, unmarried or separated women, and those with a high school education or less (Table 2). In contrast, smoking status did not differ significantly across different ages in the range 20–44 (*p* = 0.091) and income (*p* = 0.245) groups.

Next, we analyzed the association between folate insufficiency and serum cotinine concentration among these pregnant women. Logistic regression analyses revealed that the odds of RBC folate insufficiency were higher among those with higher serum cotinine levels. Specifically, for each 12.5 ng/mL increase in serum cotinine concentration—equivalent to 1 mg nicotine intake [18] or the average amount of nicotine a cigarette smoker absorbs per cigarette [19]—we observed a 7% increase (95% CI: 3, 12%) in the odds of RBC folate insufficiency. This association remained significant even after adjusting for sociodemographic factors, such as age, race/ethnicity, marital status, educational level, and family income (Table 3). Furthermore, pregnant women who were active smokers had nearly double the odds of experiencing RBC folate insufficiency compared to nonsmokers (adjusted OR: 1.87; 95% CI: 1.10, 3.19) (Table 3). However, among pregnant women heavily exposed to secondhand smoke, we did not observe this significant association.

To quantify robustness to unmeasured confounding, we computed *E*-values based on the survey-weighted risk-ratios. Compared with nonsmokers, active smokers had an adjusted risk ratio (RR) of 1.31 (95% CI: 1.12–1.54); the corresponding *E*-value was 1.95 (Table 5). For context, the RR of folate insufficiency for age 25–34 compared to age 20–24 was 0.78 (95% CI: 0.59–1.03), for age 35–44 compared to age 20–24 was 0.67 (95%CI: 0.45–1.00), for race/ethnicity (non-Hispanic Black *vs.* non-Hispanic White) was 0.91 (95%CI: 0.47–1.75), for race/ethnicity (Mexican American *vs.* non-Hispanic White) was 0.82 (95%CI: 0.64–1.05); for race/ethnicity (other Hispanic *vs.* non-Hispanic White) was 1.30 (95%CI: 1.07–1.59); for race/ethnicity (other race/multiracial *vs.* non-Hispanic White) was 0.54 (95%CI: 0.34–0.88); for marital status (married *vs.* unmarried) was 1.27 (95%CI: 0.97–1.66); for educational level (≤ high school *vs.* ≥ some college) was 0.73 (95%CI: 0.60–0.89); and for family income-to-poverty ratio (<1.0 *vs.* ≥1.0) was 0.79 (95%CI 0.63–1.01). The RR of active smoker for age 25–34 compared to age 20–24 was 0.49 (95% CI: 0.26–0.90), for age 35–44 compared to age 20–24 was 0.57 (95%CI: 0.28–1.18), for race/ethnicity (non-Hispanic Black *vs.* non-Hispanic White) was 2.25 (95%CI: 0.46–11.07), for race/ethnicity (Mexican American *vs.* non-Hispanic White) was 5.26 (95%CI: 2.17–12.75); for race/ethnicity (other Hispanic *vs.* non-Hispanic White) was 6.83 (95%CI: 3.13–14.89); for race/ethnicity (other race/multiracial *vs.* non-Hispanic White) was 2.34 (95%CI: 0.70–7.87); for marital status (married *vs.* unmarried) was 3.95 (95%CI: 2.27–6.87); for educational level (≤ high school *vs.* ≥ some college) was 0.38 (95%CI: 0.22–0.66); and for family income-to-poverty ratio (<1.0 *vs.* ≥1.0) was 0.64 (95%CI: 0.38–1.06). Additionally, we performed a subset analysis (*n* = 638) as data on dietary folate intake were not consistently available for all included NHANES cycles, and its inclusion would have substantially reduced the sample size in the primary models. In this subset, the RR of smoking for one standard deviation increase in folate intake was 0.85 (95% CI: 0.62–1.16), and the RR of RBC folate insufficiency for one standard deviation increase in dietary folate intake was 0.98 (95% CI: 0.85–1.15); all analyses were adjusted for the effects of other covariates.

We measured the folate insufficiency burden associated with smoking during pregnancy in the U.S. The results showed that 5.1% (95% CI: 3.4, 7.0%) of RBC folate insufficiency in pregnancy in the U.S. was associated with active smoking, and this fraction was 3.6% (95% CI: 1.4, 6.4%) after adjusting for sociodemographic factors (Table 4). These results suggested that in the U.S., ~3.6% (95% CI: 1.4, 6.4%) of cases of RBC folate insufficiency among pregnant women are due to cigarette smoking.

## Discussion

In 1988, the U.S. mandated folic acid fortification and augmentation in cereal-grain products to supplement the diets of women of reproductive age to reduce the incidence of neural tube defects. There has been evidence of a slight decrease in blood folate concentrations in the U.S. population between 1999 and 2010 and a slight increase in overall blood folate concentrations between 2011 and 2016 [20]. Our study showed that a significant proportion of pregnant women in the U.S. who are non-Hispanic Black and have an education level of high school or less did not have optimal RBC folate concentrations. These findings are consistent with a previous study in the Boston Birth Cohort, which found that the highest rate of folate deficiency occurred in poor black mothers [4]. This prevalence of folate insufficiency had similar demographic characteristics to the previously reported prevalence of neural tube defects in the U.S. [21,22]. Since RBC folate indicates long-term folate status, whereas serum folate indicates short-term folate status [23], RBC folate screening during pregnancy measures the amount of folate stored in the maternal body and also improves the detection rate of folate insufficiency in pregnancy.

The National Pregnancy Risk Assessment Monitoring System (PRAMS) reported that the prevalence of smoking before, during, and after pregnancy has decreased from 2000 to 2020, and in 2021, 12.1% of women smoked before pregnancy, 5.4% smoked during pregnancy, and 7.2% smoked postpartum [24]. Although smoking prevalence has declined in high-income countries over the past few decades, smoking during pregnancy remains a public health issue in the U.S. [25]. Our study observed sociodemographic differences in smoking prevalence among pregnant women in the U.S. Both the highest rate of heavy exposure to secondhand smoke and the highest rate of active smoking were identified in pregnant women who were non-Hispanic black, unmarried or separated, and with an education level of high school or less. These findings are consistent with previous epidemiological studies. Azagba et al. [10] found that the prevalence of smoking during pregnancy in the U.S. was highest among women with a high school diploma. According to a study on nonsmoking pregnant women in New York City, African American and Black Hispanic women were at the highest risk of heavy exposure to secondhand smoke [26]. However, different from the findings of Kondracki’s study [27], which indicated that non-Hispanic whites were one of the strongest predictors of smoking anytime during pregnancy, our study found that non-Hispanic black pregnant women had the highest rate of active smoking during 2003–2020. Of course, the periods covered in both studies differ; the former work used the 2016 National Center for Health Statistics (NCHS) Natality File on live births.

Our results showed that pregnant women who smoked or were exposed to secondhand smoke shared similar sociodemographic characteristics with those who had folate insufficiency in pregnancy. The results also indicated that pregnant active smokers exhibited a statistically significantly higher risk of RBC folate insufficiency compared to non-smoking pregnant women. Although non-Hispanic blacks metabolize cotinine slower than non-Hispanic whites [28], our findings indicate that this metabolic difference does not negate the association between cotinine level and RBC folate concentration. Logistic regression analyses revealed that this association remained statistically significant even after adjusting for race/ethnicity, suggesting that the observed relationship is not solely due to differences in cotinine metabolism. Although the optimal serum cotinine cut-off values to distinguish smokers from non-smokers may vary by race/ethnicity [28], we adopted the CDC/NHANES-recommended threshold which has been widely used in U.S. population-based studies due to its established validity in large, multi-ethnic cohorts [29,30]. Additionally, our study showed that tobacco smoking is significantly associated with RBC folate concentrations which serve as maternal folate reserves, suggesting a potential interaction between nicotine and folate. Folate is connected to congenital malformations, such as neural tube defects by its involvement in epigenetic regulation [31]. Nicotine has been demonstrated to induce neural tube defects and other embryonic malformations via oxidative stress in animal models [32]. Although the molecular mechanisms underlying the nicotine-folate interaction in embryonic malformations remain unclear, the mechanisms of their interaction have been elucidated in areas, such as islet cell mitochondrial dysfunction [33], cardiac injury [34], anxiety and depressive-like behavior [35], and memory impairment [36]. Given this gap, we believe that investigating these mechanisms will be a valuable direction for future scientific research, potentially providing insights into methods for addressing nicotine-folate interactions in embryos.

While our analysis highlights a significant association between smoking and RBC folate insufficiency among women of reproductive age, the calculated population attributable fraction (PAF) of 3.6% indicates that smoking accounts for only a modest proportion of the overall folate insufficiency burden in this population. This relatively low PAF suggests that, although smoking cessation interventions should undoubtedly be promoted to mitigate this modifiable risk factor and its broader health impacts potentially on the fetus and newborns, such efforts alone are unlikely to substantially reduce the high prevalence of folate insufficiency, which affects nearly half of the women in our study. Notably, smoking during pregnancy is more prevalent among disadvantaged populations and is often associated with other unhealthy lifestyle factors, such as poor dietary habits or poor nutrition [37,38], which may further exacerbate folate insufficiency. Meaningful population-level improvements will therefore require comprehensive strategies, including improving adherence to folate supplementation as the universal folate supplementation recommended in some countries and addressing structural and socioeconomic determinants of nutrition, such as access to fortified foods or healthcare. This finding underscores the need for multifaceted public health strategies that address other dominant contributors, such as dietary inadequacies, socioeconomic factors, or access to supplementation. By acknowledging the limited attributable impact of smoking, our results emphasize the importance of comprehensive folate fortification programs and nutritional education to achieve meaningful reductions in insufficiency rates at the population level.

**Figure 1.**
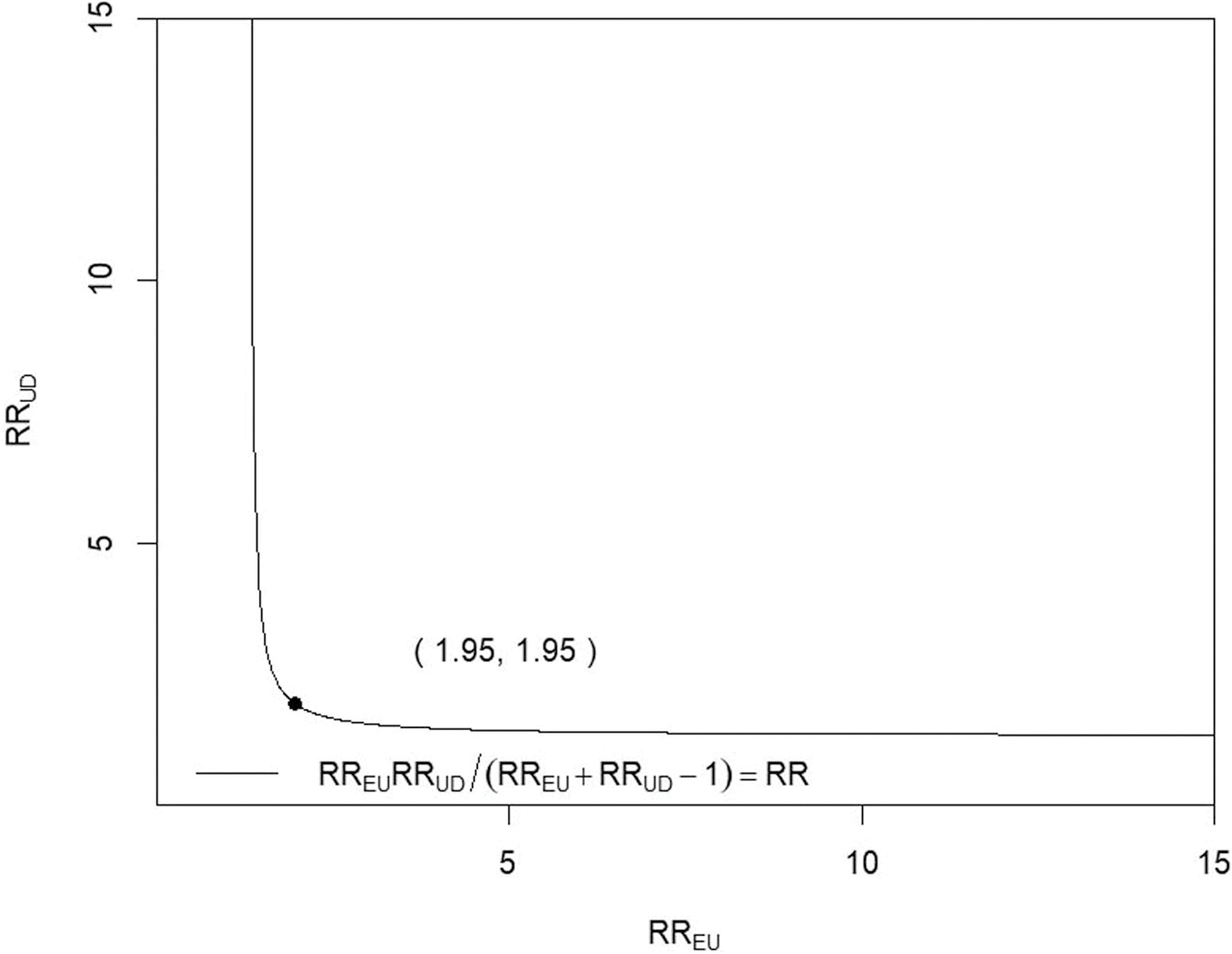
A plot of different combinations of RR_UD_ and RR_EU_ that are required to explain away the observed relative risk. Abbreviations. RR_EU_, strength of association between the unmeasured confounder and smoking; RR_UD_, strength of association between the unmeasured confounder and RBC folate insufficiency.

There are some limitations to this study. This is a cross-sectional observational study, and this design inherently limits its ability to establish causal relationships. However, to establish a cause-effect relationship, a clinical experiment is necessary. On the other hand, conducting clinical experiments with pregnant human subjects is difficult, if not impossible. Therefore, we have relied on this observational study and assessed the observed associations’ sensitivity to potential unobserved confounders or confounders not included in our analysis. We performed the sensitivity analysis by calculating the *E*-value [17]. It is suggested that an unobserved confounder would need to be associated with both folate insufficiency and smoking greater than the *E*-value on the risk ratio scale (Table 5) to explain away the observed estimates completely. Our estimated RR 1.31 implies that the risk of folate insufficiency is 31% higher among the smokers than the non-smokers after adjusting for the effect of the covariates. The *E*-value of 1.95 (Figure 1) signifies that the RR between folate insufficiency and any given covariate (not included in the analysis) is larger than 1.95, and the RR between smoking and the covariate is larger than 1.95, negating the association between smoking and folate insufficiency. Seeing none of the confounding variables’ RR estimates (one with folate insufficiency and the other with smoking) simultaneously exceeding 1.95, we conclude that the two RR estimates for any unobserved covariate are not likely to simultaneously exceed 1.95, which implies the association between smoking and folate insufficiency is not likely an artifact of the effect of the confounding variables.

One such potential confounder is supplementary folate intake. Given the large proportion of missing data, we conducted a subset analysis and the results showed that the RRs are nowhere near 1.95, so dietary folate intake is unlikely to reduce the observed association to the null. Prior work has shown that even after correction for dietary folate intake, smokers still had substantially lower plasma folate, erythrocyte folate, and buccal mucosal cell folate compared with nonsmokers [39]. Specifically [39], it noted that after correction for dietary folate intake, sex, and alcohol consumption, the plasma, red blood cell, and buccal mucosal cell folate concentrations were 20.3, 32.2, and 50.5% lower in the smoking group compared to non-smoking group, all with *p*-value smaller than 0.05. These findings indicate that smoking exerts an additional adverse effect on folate status, even after accounting for dietary folate intake. Another potential confounder is gestational age, which was not known and thus was not included in the analysis.

Although our study cannot identify the directionality of the observed association, a Norwegian randomized clinical intervention trial [40] established the directionality of this association by demonstrating that smoking decreased participants’ circulating folate concentrations, and their folate concentrations increased significantly after smoking cessation. Furthermore, a recent study from Johns Hopkins University showed that adequate maternal folate concentrations could reduce smoking-induced offspring Aryl Hydrocarbon Receptor Repressor (AHRR) gene hypomethylation by almost half [41], which demonstrated the significance of adequate maternal folate levels in protecting pregnant smokers’ offspring from birth defects.

## Conclusion

Our findings suggest that tobacco smoking is associated with insufficient folate stores in pregnancy, which may contribute to an increased risk of severe birth defects. Given the high prevalence of insufficient folate stores and the low population attributable fraction for smoking, healthcare providers should assess the folate status of all pregnant women and offer proactive screening, such as RBC folate testing. Once the prevalence of insufficient folate stores has been reduced at the population level, proactive screening for smokers should be revisited. For pregnant women who smoke or are exposed to tobacco smoke, daily folate supplementation could be especially beneficial, and reinforcing smoking-cessation support may help reduce the risk of folate insufficiency. Additionally, since higher smoking rates and folate insufficiency are more prevalent among non-Hispanic black pregnant populations with lower socioeconomic status, such measures may provide a remedy for the preventable issues of tobacco smoking and folate insufficiency prevalent in these populations, which may help address the problem of health disparities among these populations.

## Supplementary Material

Supp 1

Supplemental data for this article is available online at https://doi.org/10.1080/14767058.2025.2577231

## Figures and Tables

**Table 1.** Demographic characteristics by folate status in pregnancy (NHANES 2003–2020).^a^

	RBC folate insufficiency (*n* = 368)	RBC folate sufficiency (*n* = 412)	*p*-Value

Age			0.126
20–24	123 (56.80%)	106 (43.20%)	
25–34	210 (44.12%)	236 (55.88%)	
35–44	35 (39.16%)	70 (60.84%)	
Race			0.038
Non-Hispanic White	128 (41.55%)	189 (58.45%)	
Non-Hispanic Black	82 (64.32%)	59 (35.68%)	
Mexican American	111 (51.81%)	91 (48.19%)	
Other Hispanic	26 (47.79%)	28 (52.21%)	
Other races—including multiracial	21 (27.72%)	45 (72.28%)	
Marital status			0.159
Married/living with partner	277 (44.43%)	341 (55.57%)	
Unmarried/separated	91 (56.82%)	71 (43.18%)	
Education level			0.006
High school graduate or less	191 (56.28%)	153 (43.72%)	
College graduate or above	177 (39.80%)	259 (60.20%)	
Ratio of family income to poverty^b^			0.095
<1.0	122 (54.37%)	92 (45.63%)	
≥1.0	246 (43.74%)	320 (56.26%)	

a Percentages adjusted for sampling weights, stratum and primary sampling unit.

b Poverty-income ratio: a ratio <1 means that the income is less than the poverty level.

**Table 2.** Demographic characteristics by smoking status in pregnancy (NHANES 2003–2020).^a^

	Nonsmokers (*n* = 650)	^b^SHS exposure (*n* = 31)	Active smokers (*n* = 99)	*p*-Value

Age				0.091
20–25	172 (74.34%)	20 (8.57%)	37 (17.09%)	
25–34	390 (88.92%)	8 (2.19%)	48 (8.90%)	
35–44	88 (88.02%)	3 (1.46%)	14 (10.52%)	
Race				0.003
Non-Hispanic White	257 (82.26%)	7 (1.80%)	53 (15.94%)	
Non-Hispanic Black	98 (67.27%)	16 (14.75%)	27 (17.97%)	
Mexican American	190 (95.04%)	4 (1.94%)	8 (3.03%)	
Other Hispanic	45 (88.62%)	4 (4.77%)	5 (6.61%)	
Other races—including muti-racial	60 (92.77%)	0 (0.00)	6 (7.23%)	
Marital status				0.002
Married/living with partner	548 (90.50%)	14 (2.02%)	56 (7.48%)	
Unmarried/separated	102 (62.11%)	17 (11.06%)	43 (26.84%)	
Education level				0.008
High school graduate or less	264 (76.68%)	20 (6.04%)	60 (17.28%)	
College graduate or above	386 (91.01%)	11 (2.15%)	39 (6.84%)	
Ratio of family income to poverty^c^				0.245
<1.0	158 (79.47%)	14 (5.53%)	42 (15.00%)	
≥1.0	492 (87.10%)	17 (3.10%)	57 (9.79%)	

a Percentages adjusted for sampling weights, stratum, and primary sampling unit.

b SHS exposure: heavy exposure to secondhand tobacco smoke.

c Poverty-income ratio: a ratio <1 means that the income is less than the poverty level.

**Table 3.** Association between RBC folate insufficiency and cotinine-derived smoking status.

	Odds ratio (95% CI)	*p*-Value	^a^Odds ratio (95% CI)	*p*-Value

Cotinine concentration (12.5-ng/ml increase)^b^	1.07 (1.03, 1.12)	0.004	1.06 (1.003, 1.113)	0.041
Smoking status				
Nonsmoker	Ref	–	Ref	–
SHS exposure^c^	1.51 (0.42, 5.48)	0.502	0.80 (0.17, 3.85)	0.709
Active smoker	2.22 (1.82, 2.71)	<0.001	1.87 (1.10, 3.19)	0.030

a Model adjusted for age, race/ethnicity, marital status, educational level, and family income.

b Odds ratio for a 12.5-ng/ml increase in serum cotinine concentration.

c SHS exposure: heavy exposure to secondhand tobacco smoke.

**Table 4.** Population attributable fractions of folate insufficiency associated with cotinine-derived smoking status among pregnant women in the US.

	PAF% (95% CI)	^a^PAF% (95% CI)

Non-smoker	Ref	Ref
Active smoker	5.1 (3.4, 7.0)	3.6 (1.4, 6.4)

a PAF adjusted for age, race/ethnicity, marital status, educational level, and family income.

**Table 5.** Overall risks of folate insufficiency in pregnancy for nonsmokers and smokers.

	Risk ratio (95% CI)	*E*-value	^a^Risk ratio (95% CI)	*E*-value

Non-smoker	Ref	–	Ref	–
Active smoker	1.44 (1.32, 1.57)	2.24	1.31 (1.12, 1.54)	1.95

a Model adjusted for age, race/ethnicity, marital status, educational level, and family income.

## Data Availability

All relevant data can be obtained from the NHANES website: https://www.cdc.gov/nchs/nhanes/index.htm.
