# Supplementary material for: Nicotine consumption and folate insufficiency in pregnancy: a population-based cross-sectional study
Source: J Matern Fetal Neonatal Med. Author manuscript; Available in PMC 2026 Jan 12. (PMC12794398; doi:10.1080/14767058.2025.2577231)
Supplement: Supp 1 [file NIHMS2120157-supplement-Supp_1.pdf]

Supplementary materials for "Nicotine consumption and folate insufficiency in pregnancy: a population-based cross-sectional study" by Huang et al.

1 Odds Ratio (OR)

The model is defined as: $\operatorname{logit}(\mathbf{p}) = \mathbf{X}\boldsymbol{\beta}$ (1)

where $\mathbf{p} = \begin{bmatrix} p_1 \\ \vdots \\ p_n \end{bmatrix}, \quad \mathbf{X} = \begin{bmatrix} \mathbf{x}_1^\top \\ \vdots \\ \mathbf{x}_n^\top \end{bmatrix}, \quad \mathbf{x}_i = \begin{bmatrix} 1 \\ X_i \\ \mathbf{Z}_i \end{bmatrix}, \quad \boldsymbol{\beta} = \begin{bmatrix} \beta_0 \\ \beta_1 \\ \boldsymbol{\beta}_2 \end{bmatrix}.$

For every individual:

$$\operatorname{logit}(p_i) = \beta_0 + \beta_1 X_i + \boldsymbol{\beta}_2^\top \mathbf{Z}_i, \qquad X_i \in \mathbb{R},\ \mathbf{Z}_i \in \mathbb{R}^q,\ \boldsymbol{\beta}_2 \in \mathbb{R}^q$$

where $p_i = P(Y_i = 1 | X_i, \mathbf{Z}_i)$ is the probability of the response, X_i is the exposure variable, $\mathbf{Z}_i$ is the vector of covariates, and $\boldsymbol{\beta}_2$ is the corresponding coefficient vector.

Maximize the weighted log-likelihood function:

$$\ell(\beta) = \sum_{i=1}^{n} w_i \left[y_i \log(p_i) + (1 - y_i) \log(1 - p_i) \right],$$

where w_i is the weight, y_i is the observed response (0 or 1), and $p_i = \operatorname{expit}(\eta_i) = \frac{\exp(\eta_i)}{1+\exp(\eta_i)}$ with $\eta_i = \beta_0 + \beta_1 X_i + \boldsymbol{\beta}_2^\top \mathbf{Z}_i$.

$$\frac{\partial \ell_w}{\partial \boldsymbol{\beta}} = \sum_{i=1}^{n} w_i \left(\frac{y_i}{p_i} - \frac{1 - y_i}{1 - p_i} \right) \frac{\partial p_i}{\partial \boldsymbol{\beta}}, \qquad \text{where } \frac{\partial p_i}{\partial \boldsymbol{\beta}} = \frac{\partial p_i}{\partial \eta_i} \frac{\partial \eta_i}{\partial \boldsymbol{\beta}} = p_i (1 - p_i)\, X_i$$

Therefore, $\dfrac{\partial \ell_w}{\partial \boldsymbol{\beta}} = \displaystyle\sum_{i=1}^{n} w_i\, X_i\, (y_i - p_i).$

The parameters β are estimated by solving the weighted score equations:

$$\sum_{i=1}^{n} w_i\, X_i\, (y_i - p_i) = \mathbf{0} .$$

$$\widehat{\mathrm{OR}}_{\text{adjusted}}(g \,\text{vs}\, \text{ref}) = \exp(\hat{\beta}_g) \qquad \text{(g-th level of categorical exposure vs. reference level exposure)} .$$

$$\widehat{\mathrm{OR}}_{\text{adjusted}}(\Delta X) = \exp\big(\hat{\beta}_1\, \Delta X\big) \qquad \text{(per } \Delta X \text{ increase in the continuous exposure)} .$$

2 Risk Ratio (RR)

For every individual:

$$p_{1i} = P(Y_i = 1 \mid X = 1, Z_i;\, \hat{\beta}) = \frac{\exp\big(\hat{\beta}_0 + \hat{\beta}_1 + \hat{\beta}_2^\top Z_i\big)}{1 + \exp\big(\hat{\beta}_0 + \hat{\beta}_1 + \hat{\beta}_2^\top Z_i\big)}.$$

$$p_{0i} = P(Y_i = 1 \mid X = 0, Z_i;\, \hat{\beta}) = \frac{\exp\big(\hat{\beta}_0 + \hat{\beta}_2^\top Z_i\big)}{1 + \exp\big(\hat{\beta}_0 + \hat{\beta}_2^\top Z_i\big)}.$$

where $Y_i \in \{0,1\}$ is the binary outcome, $X_i \in \{0,1\}$ is the binary exposure indicator (1 = exposed, 0 = unexposed), and $\hat{\boldsymbol{\beta}}$ are obtained from the logistic regression model (1) .

$$\bar{p}_1 = \frac{\sum_{i=1}^{n} w_i\, p_{1i}}{\sum_{i=1}^{n} w_i}, \qquad \bar{p}_0 = \frac{\sum_{i=1}^{n} w_i\, p_{0i}}{\sum_{i=1}^{n} w_i}.$$

$$\widehat{\mathrm{RR}}_{\text{adjusted}} = \frac{\bar{p}_1}{\bar{p}_0} \qquad \text{(population-averaged effects)} \tag{2}$$

3 E-values

$$\text{E-value}_{\text{est}} = \begin{cases} \widehat{RR}_{\text{adjusted}} + \sqrt{\widehat{RR}_{\text{adjusted}}(\widehat{RR}_{\text{adjusted}} - 1)} & \text{if } \widehat{RR}_{\text{adjusted}} > 1, \\ \frac{1}{\widehat{RR}_{\text{adjusted}}} + \sqrt{\frac{1}{\widehat{RR}_{\text{adjusted}}}\left(\frac{1}{\widehat{RR}_{\text{adjusted}}} - 1\right)} & \text{if } \widehat{RR}_{\text{adjusted}} \leq 1. \end{cases}$$

where $\widehat{RR}_{\text{adjusted}}$ is the point estimate of the adjusted risk ratio obtained above (2) .

4 Population Attributable Fraction

$$PAF_{\text{adjusted}} = \frac{\pi \times (RR_{\text{adjusted}} - 1)}{1 + \pi \times (RR_{\text{adjusted}} - 1)},$$

where π is the prevalence of the exposure in the population. RR_{adjusted} is the estimated relative risk adjusted for covariates (2) .

5 Code Availability

All analysis code is available at:
`https://github.com/DrHuang123/Nicotine-consumption-and-folate-insufficiency`.
